# Adolescents’ experience of a rapid HIV self-testing device in youth-friendly clinic settings in Cape Town South Africa: a cross-sectional community based usability study

**DOI:** 10.7448/IAS.19.1.21111

**Published:** 2016-12-23

**Authors:** Philip Smith, Melissa Wallace, Linda-Gail Bekker

**Affiliations:** ^a^ The Desmond Tutu HIV Centre, Institute for Infectious Disease and Molecular Medicine, Faculty of Health Science, University of Cape Town, Cape Town, South Africa

**Keywords:** acceptability, diagnostic tests, epidemiology, usability, HIV, self-testing.

## Abstract

**Introduction**: Since HIV testing in South African adolescents and young adults is sub-optimal, the objective of the current study was to investigate the feasibility and acceptability of an HIV rapid self-testing device in adolescents and young people at the Desmond Tutu HIV Foundation Youth Centre and Mobile Clinic.

**Methods**: Self-presenting adolescents and young adults were invited to participate in a study investigating the fidelity, usability and acceptability of the AtomoRapid HIV Rapid self-testing device. Trained healthcare workers trained participants to use the device before the participant conducted the HIV self-test with device usage instructions. The healthcare worker then conducted a questionnaire-based survey to assess outcomes.

**Results**: Of the 224 enrolled participants between 16 and 24 years of age, 155 (69,2%) were female. Overall, fidelity was high; 216 (96,4%) participants correctly completed the test and correctly read and interpreted the HIV test result. There were eight (3,6%) user errors overall; six participants failed to prick their finger even though the lancet fired correctly. There were two user errors where participants failed to use the capillary tube correctly. Participants rated acceptability and usability highly, with debut testers giving significantly higher ratings for both. Younger participants gave significantly higher ratings of acceptability.

**Conclusions**: Adolescents and young adults found HIV self-testing highly acceptable with the AtomoRapid and they used the device accurately. Further research should investigate how, where and when to deploy HIV self-testing as a means to accompany existing strategies in reaching the UNAIDS goal to test 90% of all individuals worldwide.

## Introduction

Access to regular HIV testing and knowledge of one’s status is the gateway to HIV prevention and treatment and thus a public health imperative [1,2]. The UNAIDS launch of the 90-90-90 campaign has set an ambitious goal of reaching 90% of people worldwide with HIV testing [3]. This is in an effort to ensure all those who are positive are offered life saving antiretroviral therapy. What is more, knowledge of HIV status has been associated with a reduction in sexual risk behavior and improved linkage to HIV care and treatment services [4–6].

Until recently, HIV counselling and testing (HCT) has been accessible from primary healthcare facilities such as clinics and day hospitals. However, the majority of adolescents and young people living in Sub-Saharan Africa have not tested and do not know their HIV status [7,8]. Adolescents do not or cannot access healthcare services including HIV testing due to perceived or actual barriers of accessibility, overburdened health systems, lack of confidentiality and stigma [9–12]. Locating HIV testing services outside of traditional health facilities may provide more accessible, less stigmatizing opportunities for young people to test. Community based testing, including mobile testing, home testing and self administered tests may further facilitate testing in the home or other more acceptable venues and contribute towards the first 90 of the 90-90-90 goals.

Commercially, there are a plethora of “gold standard” lateral flow (LF) blood test kits and oral/buccal mucosal “rapid” tests which test for HIV antibodies giving an almost immediate, point of care diagnosis of HIV infection. Traditionally these have been administered by trained healthcare workers or counselors. Self-testing kits are similarly designed, but come with detailed user instructions so that an individual may (1) collect his or her own blood specimen/buccal fluid specimen (2) perform a simple rapid HIV test and (3) interpret the result in private [13,14]. The HIV self-testing device that tests buccal fluid and is known as OraQuick has been found to be highly acceptable by adult and high risk groups [15,16]. While most research in HIV self-testing has been conducted with buccal fluid, one study investigated the use of a blood based HIV test (Abbott Determine 1/2) and recommended deferring self-testing with the device due to a failure in compliance in one or more steps and the high rates (12%) of test result misinterpretation [13].

Although saliva-based HIV testing may be easier for self-testing and does not involve universal precautions, there are documented issues about sensitivity in this setting [17]. While blood-based assays are less expensive, more available and considered the norm, in order for blood sample self-administered tests to be safe, acceptable, and widely used, ease of use, fidelity and reliability will be critical factors to be assessed prior to any accreditation. Atomo Diagnostics Pty Ltd. has developed an “all-in-one” LF blood test device known as the AtomoRapid™ Professional Device. This test differs from traditional HIV tests because the lancet, the capillary blood collection tube, and the test strip are all contained in one device. The objective of the study was to investigate usability, acceptability and fidelity of the AtomoRapid device as an HIV self-test among adolescents and young people in high HIV burden communities in Cape Town, South Africa.

## Methods

**Figure 1.**
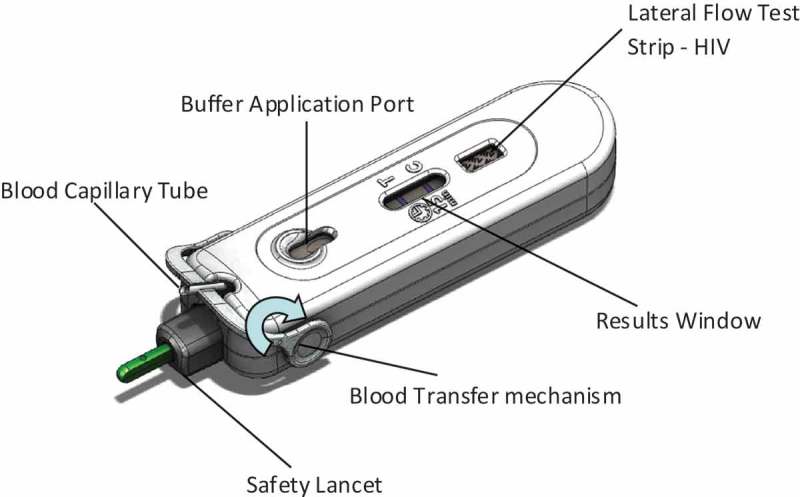
AtomoRapid device.

The HIV testing device under examination in this study was the MicroRapid Professional Version 1 HIV Assay MRLF (Part # MRLF-A-10, revision B) device known as AtomoRapid. The AtomoRapid device uses the Advanced Quality Rapid Anti-HIV (1&2) test. The Elisa and Western Blot reference tests indicated 100% specificity for 150 confirmed negative samples, and 100% sensitivity for 150 confirmed positive samples [18]. The diagnostic performance of the device has been shown by the South African National Institute for Communicable Diseases and communicated via email after testing in standard clinic environments following standard procedures and techniques for LF blood testing (P. Dabula, [patience.dabula@nhls.ac.za], letter, 10 July, 2013). As shown in Figure 1, the device contains an automatically retracting safety lancet, a blood collection capillary tube that flips over to deposit a blood sample onto the lateral flow test strip with its result indicator all in one self-contained device.

### Participants

A convenience sample of self-presenting adolescents and young adults between the ages of 16 and 25 years, routinely attending the Desmond Tutu Youth Centre Clinic (DTYC) and the Desmond Tutu HIV Foundation’s Tutu Tester Mobile clinic, were recruited into the study over a six-month period between May and October 2014. The participants differed from the normal clinic population in that people between 16 and 25 were recruited because they are at increased risk of HIV infection and they are less likely to test for HIV at a traditional clinic facility. These community based services provide healthcare in under-resourced communities and have both been described elsewhere [19,20]. Point of care HIV testing is offered free of charge as part of the service package in both settings. Participants who were willing to provide informed consent for the HIV self-test and participate in the research study were included. The study was reviewed and approved by the University of Cape Town Health Science Research Ethics Committee. HIV testing does not require parental consent over the age of 12 years in South Africa. On these grounds parental consent was waived by the Ethics Committee for participants aged 16 and 17 years old. Appropriate counseling, care and referral support was offered to all testers on the Tutu Tester mobile services and at the DTHF Youth Centre clinic.

### Procedure

Participants were met by a trained and experienced healthcare practitioner (HCP), an HCT counsellor or nurse. After informed consent procedures, the participants were trained to use the AtomoRapid device. The training session included an explanation and a demonstration of the device and included study participants reviewing the pictorial Instructions For Use (IFU) contained in the device packaging. This training took on average five minutes and was conducted by a counselor. Participants then completed an HIV self-test with the AtomoRapid device under the supervision of, but without assistance from the HCP. If the participant asked for assistance, the HCP asked the participant to continue until the test was completed. The steps of use for the device included the following: Participants selected and wiped a finger with an alcohol swab, they then released the sterility tab on the lancet and applied the lancet pad to their finger and the lancet was triggered by applied pressure. They were then required to milk a blood bubble on the finger pad and allow collection of a 50uL sample into the capillary tube. The tube was then flipped over onto the test strip. Participants were required to apply buffer solution to the strip and wait 15 minutes before reading and interpreting the test result. The HCP then interviewed the participant using a questionnaire rating the usability and acceptability of the device. After completing the questionnaire, participants answered questions about their demographics.

### Outcome measures

The questionnaire administered by the HCP assessed usability and acceptability of the device on a likert type scale with ratings ranging from 1–5 on both continuous variables. Usability was defined as the ease of using the device and was operationalised by asking participants to rate the ease of use of the device on eight domains. Acceptability was defined as preference for using the device and was operationalised by asking participants to rate their preference for self-testing. A score of 5 indicated a better rating in all cases except for the question that asked whether the participant would self-test again with this device (5 indicated very unlikely). The scale was designed to measure the ease of use of each step of using the device. The subscales for usability were combined to create a composite score for usability [21,22]. The same process was followed to create an acceptability score. The HCP recorded any process errors relating to the steps of use. The total number and types of errors, including result interpretation, were recorded. Each step was recorded as correctly or incorrectly completed.

As per clinic standard operating procedure, participants who tested HIV negative received risk reduction counseling. Participants who tested HIV positive were referred to the clinic nurse for assessment for treatment, including a rapid CD4 count. Newly diagnosed individuals were referred with a comprehensive nurse’s letter to their preferred clinic facility for ongoing treatment.

### Analysis

Statistical analyses were conducted using version STATA 14 (Stata Corporation, College Station, TX) at the 5% level of siginificance. The study was descriptive and exploratory, making use of inferential statistics. There were no a priori hypotheses. Demographics were entered as categorical variables. The F test, X^2^ and Fisher exact tests were used to analyse outcomes for categorical variables. Bivariate and multivariate regression was used to identify factors independently associated with usability, acceptability, and fidelity. Statistically significant factors at *p* ≤ 0.05 in the bivariate analysis were entered into a multivariable analysis. Subsequently, a stepwise approach was used to explore interactions between factors associated with outcome variables.

## Results

Of the 225 participants, between the ages of 16 and 25 years (mean age 19) 224 were retained in the analysis and 69.2% were female (Table 1), which was representative of the gender split over the course of the study. One participant was excluded since no data was recorded for the participant. The sample consisted of predominantly Xhosa speaking South Africans who lived in brick and mortar housing, or formal accommodation (*n *= 141, 63.8%), with the remaining participants living in shanty houses. There were 45 (20.1%) participants who reported no prior HIV test and therefore the AtomoRapid self-test was their first experience of HIV testing.

**Table 1.** Participant demographics and bivariate regressions for acceptability and usability

	n (%)	meanª	p*	n	mean†	p∆
**Total**	**224 (100)**	**4.2**		222	**4.0**	
**Age (*m* = 19,42)**	224		0.002			0.641
**Sex**			0.499			0.028
Female	155 (69.20)	4.2		153	3.9	
Male	69 (30.80)	4.1		69	4.1	
**Employment**			0.301			0.049
Unemployed	206 (91.96)	4.2		204	3.9	
Employed	18 (8.04)	4.0		18	4.2	
**Income**			0.056			0.292
Income	25 (11.16)	3.9		25	4.1	
No Income	199 (88.84)	4.2		197	4.0	
**Education**			0.137			0.010
Primary School	13 (5.86)	3.8		13	3.8	
High School	193 (86.94)	4.2		192	4.0	
College/University	16 (7.21)	4.2		15	4.2	
**Dwelling type**			0.327			0.000
Formal housing	141 (63.80)	4.2		139	4.1	
Informal housing	80 (36.20)	4.1		80	3.8	
**Marital Status**			0.110			0.270
Single	218 (97.76)	4.2		216	4.0	
Cohabiting	1 (0.45)	4.0		1	3.4	
Married	4 (1.79)	3.6		4	4.3	
**Ever Tested**			0.000			0.000
Never Tested	45 (20.09)	4.5		45	4.2	
Tested Before	179 (79.91)	4.1		177	3.9	

ªMean Acceptability scores

*Bivariate regression for Acceptability

†Mean Usability scores

∆Bivariate regression for Usability

*Not all columns total to 224 due to missing data*

### Fidelity

All but eight (3.6%) participants correctly completed, read and interpreted their test result. Out of the eight, there was one user error where a participant failed to fill the capillary tube sufficiently. There was a single device failure where the blood transfer mechanism broke while the participant flipped the capillary tube over to the test strip. Additionally, in six instances the participant failed to correctly pierce their finger with the lancet, although the device lancet had fired correctly. When the test was repeated all eight participants used the device correctly. Table 2 below summarizes errors of use.

**Table 2.** User device errors

Type of error
6 Difficulties firing /activating the safety lancet (with one hand)
1 Difficulties in blood Collection
1 Adequacy of collected blood volume (tube over /under filled)

### Usability

Overall, the mean score for the usability (or ease of use) of the device was 4 (median 3.9) out of a maximum rating of 5 (Table 3) where 47% of participants scored usability 4 or higher. Six out of eight subscales of usability were rated above 4, with the exception of lancet use (3.7) and collection tube use [4].

**Table 3.** Usability and acceptability rating

	*n*	Median	(IQR)
**Usability (5 = highest rating)**	222	3.9	(3,7–4,4)
1. How easy was it to undertand the device training?	220	4	(4–5)
2. How easy was it to understand the device instructions for use?	221	4	(4–5)
3. How comfortable was it to use the device?	221	4	(4–5)
4. How easy was it to activate the lancet with one hand?	222	4	(3–5)
5. How easy was it to identify when the blood collection tube was full?	222	4	(4–5)
6. How easy was it to apply the buffer solution?	220	4	(4–5)
7. How easy was it to read the test result?	220	4	(4–5)
8. How easy was it to interpret the T and Cmarkers on the device?	220	4	(4–5)
**Acceptability**	221	4.3	(3,7–5)
1. Preference for self-testing (5 = high preference)	219	4	(3–5)
2. Put off self-testing (5 = not put off)	221	4	(3–5)
3. Likelihood of telling others about HIV self-testing	217	5	(4–5)

*Not all columns total to 224 due to missing data*

### Acceptability

Participants mean acceptability of the device was 4.18 out of 5 (median 4.3). Participants generally reported preference for self-testing (74,89%) over traditional HCT, and 72,5% of participants reported that they had not been put off of self-testing. The majority of participants stated that they would tell others about self-testing (89,86%).

There were 19 participants who stated that they had had a bad experience with self-testing. Most participants (*n *= 12) who reported a bad experience said that they found it distressing to prick themselves. Six participants stated that they disliked testing themselves, while one participant did not give a reason. There were no reports of bad experience among the 45 participants who had not previously tested for HIV.

In a multivariate linear regression model (Table 4), dwelling type, education level, and whether the participant had ever tested for HIV were significantly associated with usability. There was a small, but significant difference between those living in formal dwellings and those in informal dwellings in terms of their average usability scores (0.15 *p* = 0.02). Those with higher education levels gave higher usability ratings and with every unit increase in education, usability increased by 0,10 (*p* = 0.005). Debut testers gave higher usability ratings for the device, where the average difference between those who had previously tested and debut testers was −0,30 (*p* = 0.000). Age, marital status, employment, and income had no significant effect on usability. Males rated both the usability of the instructions and the ease of reading the test result higher than females

**Table 4.** Multivariate regression models for acceptability and usability

	Estimate	95% CI	*p* value
**Determinants of Usability**			
Usability R2 = 0.15			
Ever tested	−.30	(-.45, -.15)	0.000
Education	.10	(.03, .18)	0.005
Dwelling type	.17	(.04, .30)	0.009
Constant co-efficient	3.75	(3.48, 4.02)	0.000
**Determinants of Acceptability**			
Acceptability R2 = 0.08			
Ever Tested	−.36	(-.62, -.12)	0.004
Age	−.05	(-.10, -.01)	0.026
Constant co-efficient	5.46	(4.61, 6.31)	0.000
**Usability Determinants of Acceptability**			
Usability R2 = 0.10			
Lancet use	.16	(.07, .24)	0.012
Buffer use	.17	(.04, .30)	0.000
Constant co-efficient	2.89	(2.32, 3.47)	0.000

### Acceptability

In the multivariate regression model, age and whether the participants had previously tested were significantly associated with acceptability (Table 4). Younger participants were more likely to give higher acceptability ratings and each year increase in age was associated with a 0,05 (*p* = 0.026) reduction in acceptability. Similarly, debut testers gave higher acceptability ratings where the average difference between those who had previously tested and debut testers was 0,36 (*p* = 0.004). No other demographic variables had a significant association with acceptability.

### Acceptability and usability scale items

Results confirmed that acceptability was significantly associated with good ratings of lancet use and buffer use (Table 4). Higher usability ratings for the finger prick lancet were associated with higher acceptability scores. In the multivariate regression model for the effect of items in the usability scale, these items were closely associated: for every unit increase in lancet usability, acceptability increased by 0.16 (*p* = 0.012). With every unit increase for the usability of the buffer solution, acceptability increased by 0.17 (*p* = 0.000).

## Discussion

This study confirms that HIV self-testing is highly acceptable to adolescents accessing community based testing services. This is consistent with results from older adult populations and high-risk groups [14,15]. Every test result (including the 8 repeated tests) was read and interpreted correctly, showing that self-testing can be successfully used, read and interpreted by an adolescent population.

There were six instances in which the supervising healthcare worker reported that the participants did not correctly pierce their finger. These participants were reticent about pricking their own finger, did not fire the needle correctly and therefore failed to draw blood. This suggests that those who are anxious about pricking themselves may not correctly complete the finger prick and consequently may struggle to draw blood. The lancet in the AtomoRapid device retracts into the device after firing, and therefore a new device must be used if the finger prick fails. Those who failed to use the device correctly, cited lancet use as a barrier to acceptability of self-testing. Despite this, all six participants who failed on the first attempt used the lancet successfully when given a second attempt. To account for the possibility of patients failing to prick themselves, it may be useful to include an extra lancet in the package. Participants who experience ongoing difficulty in self-pricking may find other options, such as oral/buccal mucosal self-testing more acceptable.

Overall, this device and self-testing was acceptable to adolescents and young adults. Debut and younger testers rated the device more acceptable than their peers. This may be related to younger testers not having preconceived ideas about testing and the novelty of the experience may have increased enthusiasm. Conversely, those who were older and/or HIV test experienced may have had preconceived expectations different from the self-testing experience, which might have led to slightly less favourable, although by no means unfavourable, ratings of acceptability. In addition, those in formal housing and those with higher education rated usability higher. Despite this we did not find an association between dwelling type and education. It is possible that individuals living in formal dwellings may be more exposed to other sorts of technology.

While the present study demonstrated that young people can self-test with the AtomoRapid, this study did not explore the environment in which self-testing can be safely undertaken by adolescents. In this case, the testing was done in a supportive environment where health care professionals were on hand to provide support and counseling. The safe deployment of devices to individuals to enable testing in private should be further explored. The results of this study demonstrate that self-testing may encourage earlier case-finding in the adolescent population, with the possible benefit of reducing infections [23].

## Conclusions

Adolescents in South Africa are a vulnerable population at significant risk of HIV acquisition. HIV testing is a key component of adolescent healthcare in this region and early detection and treatment of HIV prolongs life and reduces infectiousness. Inadequate testing rates show there is still room for innovation in this population. Self-testing is a novel means to make testing more accessible, confidential and available at non-traditional venues such as pharmacies and community venues as well as in the home. As PrEP has been approved for use in South Africa, HIV self-testing with an easy to use kit would be valuable in supporting self-monitoring. However, LF blood based testing with the AtomoRapid device is not currently widely available in South Africa. While not presently commercially available, consideration of the cost is pivotal in driving HIV self-testing. This study confirms that HIV self-testing with the AtomoRapid device can be used with a high degree of fidelity and acceptability by South African adolescents and youth. Further research will define how much additional support an adolescent may require when self-testing and in which environments this could be undertaken safely. Whilst disclosure, post-test support and linkage to HIV services remain barriers to the HIV care continuum, mechanisms such as self testing may assist in realizing the first 90 of 90-90-90.
